# pSTM6-275, a Conjugative IncHI2 Plasmid of Salmonella enterica That Confers Antibiotic and Heavy-Metal Resistance under Changing Physiological Conditions

**DOI:** 10.1128/AAC.02357-17

**Published:** 2018-04-26

**Authors:** Helen Billman-Jacobe, Yuhong Liu, Ruth Haites, Tom Weaver, Lily Robinson, Marc Marenda, Mike Dyall-Smith

**Affiliations:** a Veterinary Biosciences, Faculty of Veterinary and Agricultural Sciences, University of Melbourne, Parkville, Victoria, Australia; b Asia-Pacific Centre for Animal Health, Faculty of Veterinary and Agricultural Sciences, University of Melbourne, Parkville, Victoria, Australia; c National Centre for Antimicrobial Stewardship, Peter Doherty Institute, Parkville, Victoria, Australia

**Keywords:** IncHI2, Salmonella, copper, heavy metals, integrons, plasmid-mediated resistance, silver

## Abstract

Detailed annotation of an IncHI2 plasmid, pSTM6-275, from Salmonella enterica serotype 1,4,5,12:i:- strain TW-Stm6 revealed a composite structure, including antimicrobial resistance genes on mobile genetic elements. The plasmid was thermosensitive for transfer to Escherichia coli and conferred reduced susceptibility to antibiotics, copper sulfate, and silver nitrate. Metal ion susceptibility was dependent on physiological conditions, giving an insight into the environments where this trait might confer a fitness advantage.

## TEXT

Salmonella enterica is a common enteric pathogen of humans and animals and is found in many environmental and animal reservoirs with zoonotic potential. Distinct clones of multidrug-resistant S. enterica serovar Typhimurium have emerged and dominated in succession (1, 2). A recent clone of *S*. Typhimurium (strain SO4698-09) carries a Salmonella genomic island (SGI) which contributes to enhanced resistance to copper sulfate, a common animal feed additive (3). We recently reported the genome sequence of S. enterica 1,4,[5],12:i:- strain TW-Stm6, an isolate recovered from pig feces which has the same antigenic formula, phage type, and sequence type as strain SO4698-09, and also carries the genomic island, SGI-4 (3–5). The assembled genome of TW-Stm6 comprised a 4,999,862-bp chromosome, a 4-kb MOB_Q_ plasmid (pSTM6-4), and a 275.8-kb IncHI2 plasmid (pSTM6-275). Here, we report the detailed annotation of pSTM6-275 and its genetic structure, function, and transmission of antibiotic and heavy-metal resistance genes to other bacteria.

**FIG 1:**
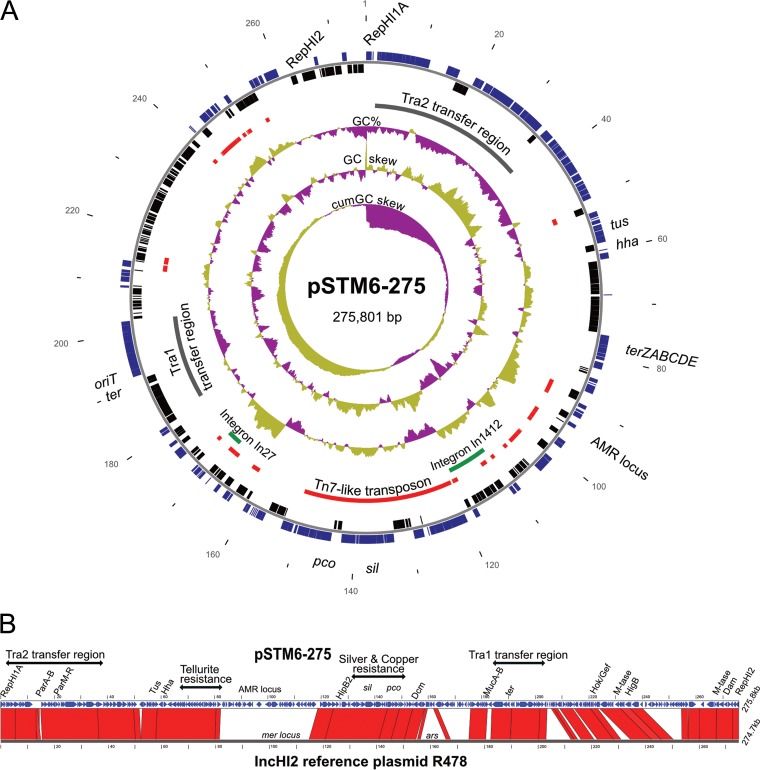
(A) Diagram of plasmid pSTM6-275 (275.8 kb). Tracks show (from outermost to center): scale in kilobase pairs; predicted coding sequences (CDS) of the top (blue) and bottom (black) strands; transposons and IS elements (red); integrons (green) and Tra regions (gray); GC%; GC-skew; and cumulative GC-skew. (B) Alignment of pSTM6-275 and the IncHI2 reference plasmid R478 (accession no. BX664015.1). Regions of nucleotide sequence similarity of ≥85% are indicated in red. Backbone regions and some of the more important proteins and loci of pSTM6-275 are indicated at the top. AMR, antimicrobial resistance.

**FIG 2:**
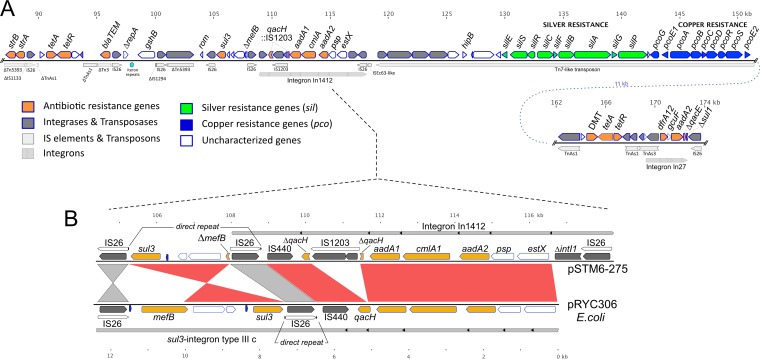
(A) Gene map of the resistance regions of pSTM6-275. Antibiotic, metal, and disinfectant resistance genes are clustered into two nearby regions, 87.1 to 151.4 kb (main sequence) and 162.1 to 173.7 kb (lower sequence connected by dotted line to the right). In each sequence, genes and operons are shown above the horizontal line, and IS elements, transposons, and integrons are displayed under the line. Integron *attC* sites are shown as triangles within integron borders. The scale at the top indicates kilobase pairs. (B) Comparison of *sul3*-integron type IIIc region of the E. coli plasmid pRYC306 (accession no. HQ875016) with the corresponding region of pSTM6-275. Nearly identical sequences are shaded red, or gray for IS*26* elements. As indicated in the diagram, the flanking 8-bp direct repeats (CTTAGGTC) of pRYC306 IS element IS*26* (nucleotides [nt] 7321 to 6502) are found split between the two leftmost copies of IS*26* in pSTM6-275. A 49-bp sequence, depicted as solid blue arrows close to the horizontal lines, occurs twice in pRYC306 (near IS*26* and near *sul3*) and once in pSTM6-275. Size scales, in kilobase pairs, are shown at the top and bottom.

Annotations were revised using EcoGene version 3 (www.ecogene.org), UniProt (www.uniprot.org), Rfam (rfam.xfam.org), BLAST (https://blast.ncbi.nlm.nih.gov/Blast.cgi), and literature searches. Plasmid typing was performed using PlasmidFinder (cge.cbs.dtu.dk/services/PlasmidFinder-1.3), the Plasmid MLST database (pubmlst.org/plasmid), and local searches of custom database sequences and IncHI typing (6). Insertion sequence (IS) elements were typed using ISFinder (https://www-is.biotoul.fr). A diagram of the 275,801-bp plasmid pSTM6-275 (accession no. CP019647.1) is shown in Fig. 1A. Many genes were clustered in functional units, such as the *ter* operon (tellurium resistance), the *sil* locus (silver efflux), the *pco* locus (copper efflux) and the transfer regions Tra1/Tra2. Tra2 has an origin of plasmid transfer, *oriT*, a potential replication terminus (*ter*) site, and a *tus* gene encoding the replication-termination protein Tus. The plasmid copy number, determined by normalizing the sequence depth relative to the chromosome, was 1.4. *In silico* typing of plasmid pSTM6-275 indicated it belonged to the IncHI2 (subtype 3) and was similar to plasmid IncHI2 reference plasmid R478 (accession no. BX664015.1) (Fig. 1B). BLASTN searches of the GenBank database (4 October 2017) failed to find any other plasmid that matched (≥99% nt identity) more than about 70% of pSTM6-275. Figure 2A depicts regions that are rich in mobile genetic elements, including IS elements, transposons, and integrons, and containing multiple resistance genes (*bla*_TEM_, *strA*, *strB*, *sul3*, *aadA1*, *aadA2*, *cmlA*, *aphA2*, and two copies of *tetA*) encoding resistance to ampicillin, streptomycin, spectinomycin, sulfonamide, trimethoprim, chloramphenicol, kanamycin, and tetracycline, respectively. Copies of *bla*_TEM_, *strA*, *strB*, *tetB*, and *sul2* occur on the chromosome in SGI-4 (5). The class I integron In27 (*dfrA12*_*gcuF*_*aadA2*_Δ*qacE*), encoding resistance to trimethoprim and streptomycin/spectinomycin, also contains a truncated *sul1* gene, disrupted by IS*26*. A second integron, In1412, classified as a novel class I integron (7), was 9,240 bp and contained the array *estX3*_*psp*_*aadA2*_*cmlA1*_*aadA1*_*qacHD4*::IS*1203* conferring resistance to streptomycin/spectinomycin and chloramphenicol (Fig. 2). A fragment of the macrolide efflux major facilitator superfamily (MFS) gene *mefB* (8) and a *sul3* element/domain (9) lie proximal to *qacH* and are flanked by divergent IS*26* elements.

A Tn*7* family transposon (32.4 kb) carrying silver and copper resistance loci, *silESRCFBAGP* and *pcoGE1ABCDRSE2* (Fig. 2A), occurs between the integrons and is delimited by inverted terminal repeats and flanking 5-bp direct repeats (Fig. 1.) Similar elements have been detected in other IncHI2 plasmids from animal-associated bacteria (10). Sil and Pco systems are composed of metal ion-binding proteins and transporters (11–13). A *sil-pco* locus with the same gene arrangement also occurs on the TW-Stm6 chromosome in SGI-4, but unlike the plasmid version, it lacks Tn*7*-like *tnsABCD* genes and inverted terminal repeats, suggesting a different evolutionary history.

Many IncHI2 plasmids are thermosensitive for transfer, with 27 to 33°C being the permissive temperature and >37°C being the nonpermissive temperature (14). Since pSTM6-275 has all the genes for proteins required for self-transmission, this function and its thermosensitive character were examined. Plasmid stability and conjugal transfer of pSTM6-275 from Salmonella to Escherichia coli DH5α were tested according to published methods (14), and the results are from two experiments performed in duplicate. Transfer occurred at 1.3 × 10^−5^ transconjugants per donor at 27°C, but no transfer was detected at 37°C. Transconjugants coinherited resistance to ampicillin, sulfonamide, streptomycin, spectinomycin, kanamycin, tetracycline, trimethoprim, and chloramphenicol, consistent with the plasmid structure. Colonies (*n* = 112) derived from a culture of the transconjugants, grown at 44°C for 24 h without antibiotic selection, retained all resistance markers, indicating that the plasmid was not thermosensitive for maintenance in E. coli. In this respect, pSTM6-275 differs from the reported phenotype of plasmid R478 (14).

The MICs of CuSO_4_ and AgNO_3_ were determined by agar dilution assays using LB agar (pH 7.2; 25 mM HEPES) (10, 12) using Oxoid AnaeroGen sachets if required. The sensitivities to of Salmonella TW-Stm6, E. coli DH5α, and two E. coli pSTM-275 transconjugants are shown in Table 1. The two transconjugants had the same MICs. The Salmonella donor had a higher AgNO_3_ MIC at 27°C (800 μM) than at 37°C (50 μM), and the MIC was not influenced by oxygen availability. The plasmid increased the MIC of AgNO_3_ for E. coli from 50 to 800 μM at 27°C; however, this effect was not seen at the higher temperature, where the MIC for all strains was 50 μM. The MIC to CuSO_4_ for E. coli was affected by oxygen. E. coli was most sensitive to CuSO_4_ under anaerobic conditions, but plasmid-bearing transconjugants were less sensitive possibly due to more efficient efflux.

**TABLE 1:** MICs of CuSO_4_ and AgNO_3_^*a*^

Substance	Strain type (strain)	MICs by temp and oxygen availability			
		37°C		27°C	
		With oxygen	Without oxygen	With oxygen	Without oxygen
AgNO_3_	Donor (Salmonella Typhimurium TW-Stm6)	50	50	800	800
	Recipient (E. coli DH5α)	50	50	50	50
	2 transconjugants	50	50	800	800
CuSO_4_	Donor (Salmonella Typhimurium TW-Stm6)	12.5	12.5	12.5	12.5
	Recipient (E. coli DH5α)	6.25	1.56	6.26	1.56
	2 transconjugants	6.25	6.25	6.25	6.25

a MICs for AgNO_3_ are given in micromolar, and those for CuSO_4_ are given in millimolar.

ST3 IncHI2 plasmids are widespread in food-producing animals (10), and despite their potential to disseminate antimicrobial resistance genes, few complete sequences have been characterized in detail. Our results suggest that the transmission of pSTM6-275 is probably restricted to outside a mammalian host given the thermosensitive nature of transfer, suggesting that it is well adapted for persistence in the environment. Furthermore, the expression of at least some of the metal resistance traits was influenced by physiological conditions. Copper metabolism in enterobacteria is complex, as several genes can be involved, including those involved in transport, oxidation, and regulation. In the present study, E. coli DH5α was sensitive to 1.56 mM CuSO_4_ without oxygen, and the acquisition of pSTM6-275 decreased the sensitivity to 6.25 mM under anaerobic conditions. E. coli can regulate copper levels by expressing chromosomal genes encoding a periplasmic copper oxidase, CueO, a cytoplasmic copper transporter, CopA, and the Cus efflux system (15). In the presence of oxygen and amino acids, copper homeostasis is achieved by CueO oxidation and CopA-mediated efflux. Cus is induced under anaerobic conditions or nutrient limitation, and E. coli CueO and Cus are not sufficient to confer Cu(I) resistance under anaerobic conditions, where nutrients are plentiful (16). Salmonella does not have a *cus*-encoded copper efflux pump and relies on CueO and CopA for copper homeostasis (15). CueO is sufficient for low-level Cu(I) tolerance and is required for virulence in mice (17).

The high level (800 μM) and temperature dependence of silver sensitivity shown by TW-Stm6 and E. coli pSTM6-275 transconjugants were unexpected. To our knowledge, this has not been previously reported, and the mechanism underlying this phenotype is unclear. It may be due to differences in thermoregulation of *sil* gene expression, the effect of temperature on the secondary structure of SilE/PcoE that alters the amount of ion binding, or changes in the outer membrane composition (18, 19).

pSTM6-275 carries a novel class I integron, In1412, that is most similar to the *sul3*-integron type IIIc region of E. coli plasmid pRYC306 (accession no. HQ875016.1). To evolve from pRYC306 to pSTM-275, one could hypothesize that (i) an IS*440* element inserted into *qacH*; (ii), an IS*26* element inserted in *mefB*, in the opposite orientation to the IS*26* element near *sul3*; and (iii) an inversion occurred via the outward-facing IS*26* elements, splitting the 8-bp direct repeats originally on the IS*26* near the *sul3* gene so they end up on two separate IS*26* copies. IS*26* is a frequently occurring and highly active insertion element in the genomes and plasmids of Salmonella spp., commonly mediating recombination events that generate new types or combinations of virulence determinants (20). Recently documented examples include novel plasmids and chromosomal loci (21–23).

The evolution of pSTM6-275 appears to be complex, and the function and regulation of many of its genes remain to be fully characterized, particularly for accessory genes, such as those involved in resistance, regulatory cross talk, and those specifying uncharacterized proteins with unknown function. Identification of other sequence type 3 (ST3) IncHI2 plasmids from human, veterinary, and environmental sources may provide further insights into the evolution of these plasmids and their role in the dissemination of resistance. This work adds to our understanding of the organization and function of an ST3 IncHI2 plasmid which may confer a fitness advantage for persistence in agricultural effluent.
